# From Trust to Choice: A Cross-Sectional Survey of How Patient Trust in Pharmacists Shapes Willingness and Vaccination Decision Control Preferences

**DOI:** 10.3390/ijerph22101525

**Published:** 2025-10-05

**Authors:** Oluchukwu M. Ezeala, Nicholas P. McCormick, Lotanna Ezeja, Sara K. Jaradat, Spencer H. Durham, Salisa C. Westrick

**Affiliations:** 1 Department of Health Outcomes Research and Policy, Harrison College of Pharmacy, Auburn University, Auburn, AL 36849, USA; 2 Department of Pharmacy Practice, Harrison College of Pharmacy, Auburn University, Auburn, AL 36849, USA

**Keywords:** SCDM, vaccinations, ACIP, trust, preference, willingness

## Abstract

Background/Objectives: The U.S. Centers for Disease Control and Prevention recommends some vaccinations using shared clinical decision-making (SCDM). SCDM recommendations are made when not every individual within a given age or risk group would benefit from vaccination, requiring collaborative discussions between patients and providers to assess risks and benefits. Pharmacists play a key role in implementing this recommendation and have frequent opportunities to engage with patients who may be eligible for SCDM-based vaccines. Because SCDM requires provider discussions to assess each patient’s eligibility for the vaccines under SCDM, trust may play a central role in the process. Trust has been suggested to affect patient’s participation in their care and their decision making preferences; however, the nature of this relationship in the context of SCDM vaccines and willingness to engage with pharmacists has yet to be investigated. As the CDC continues to expand the SCDM vaccine category, there is need to assess these. This study aimed to examine relationships between patient characteristics, trust in pharmacists, willingness to engage in SCDM, and vaccination decision control preference. Methods: Using quota sampling, cross-sectional data were collected from Alabama residents aged 18+ between February and March 2024 via a validated online questionnaire. Bivariate and multivariable logistic regression analyses were used to determine the association between trust, patient characteristics and willingness. Structural equation modeling was used to assess the direct and indirect relationships between trust, willingness and vaccination decision control preference. Statistical significance was set at *p* < 0.05. Results: A substantial portion (45.8%) of participants were unaware that certain vaccinations were based on SCDM. Multivariable logistic regression showed that race (Black vs. White, *p* = 0.001), age (25–34 vs. 18–24, *p* = 0.029), highest degree obtained (high school diploma or graduate equivalency degree vs. less than high school, *p* = 0.001; associate degree or vocational certificate vs. less than high school, *p* = 0.000; bachelor’s degree or higher vs. less than high school, *p* = 0.001), political affiliation (Democrat vs. Republican, *p* = 0.002), confidence in understanding health-related information (high vs. low, *p* =.029); moderate vs. low, *p* = 0.002), and patients’ trust in community pharmacists’ communication skills (*p* = 0.045) and benevolence (*p* = 0.001) towards their patients were significantly associated with patients’ willingness to engage in SCDM. Trust had a significant direct (*p* = 0.001) and indirect relationship (*p* = 0.000) with decision control preference through the willingness variable. Conclusions: Educational interventions are recommended to improve awareness and knowledge of SCDM vaccines among patients. Given their trusted role, pharmacists should actively build and maintain trust with patients, as this may help foster collaborative environments for discussion, encourage patient engagement in SCDM, and support more informed vaccination choices.

## 1. Introduction

The U.S. has a category of vaccination recommendations based on shared clinical decision-making (SCDM) [1]. These recommendations are made by the Advisory Committee on Immunization Practices (ACIP) of the U.S. Centers for Disease Control and Prevention (CDC) when only certain individuals within a given age or risk group would benefit from vaccination [1]. Unlike routine or catch-up vaccines, which are broadly recommended for all individuals in a defined age or risk category, SCDM vaccines require a collaborative discussion between patients and providers to determine eligibility based on individual characteristics, preferences, values, and the best available clinical evidence [1,2]. Because provider discussions are necessary to assess each patient’s eligibility for the vaccine under SCDM recommendations, trust may play a central role in this process [3].

At the time of this study (March 2024), five vaccines were recommended based on SCDM. These include meningococcal B (MenB) vaccination for adolescents and young adults aged 16–23 years, hepatitis B (HepB) vaccination for adults aged 60 years and older with diabetes mellitus, human papillomavirus (HPV) vaccination for adults aged 27–45 years, 20-valent pneumococcal conjugate vaccine (PCV20) for adults aged 65 years and older who have previously received PCV13 and PPSV23, and respiratory syncytial virus (RSV) vaccination for adults aged 60 years and older. RSV vaccination was removed from the SCDM category and transferred to the risk and routine-based category in June 2024 to simplify the recommendation process for healthcare providers, due to reported challenges and confusion in its implementation [4,5,6]. Other modifications have also been made to the SCDM category since then, such as the addition of PCV21 for adults aged ≥65 years and COVID-19 vaccination for all children and adolescents aged 6 months through 17 years, including those who are moderately or severely immunocompromised [1,7,8,9].

SCDM has long been adopted and widely explored in oncology but has not been extensively studied in the vaccination space [10,11,12,13]. Given the unique nature of vaccines compared to cancer treatments, SCDM presents distinct challenges in vaccination decisions. First, vaccines are preventive, with benefits that may not be seen until years later, making it harder for patients to perceive their immediate value. This, combined with mistrust, common misconceptions, concerns about vaccine safety and effectiveness, and uncertainty among vaccine providers about which patients should be approached for SCDM discussions, can complicate the decision-making process and potentially increase vaccine hesitancy [6,14,15,16].

Trust has been suggested to influence patients’ participation in their care and their decision-making preferences, but the nature of this relationship remains unclear in the context of SCDM vaccines and willingness to engage with pharmacists [3,17]. With limited literature on SCDM in vaccine decision-making, little is known about patients’ willingness to engage in these discussions, whether their preferences align with the collaborative nature of SCDM, and how trust in providers may shape the process. Exploring factors that influence the SCDM process for vaccines may provide insights for its implementation and suggest ways for further improvement.

**Figure 1:**
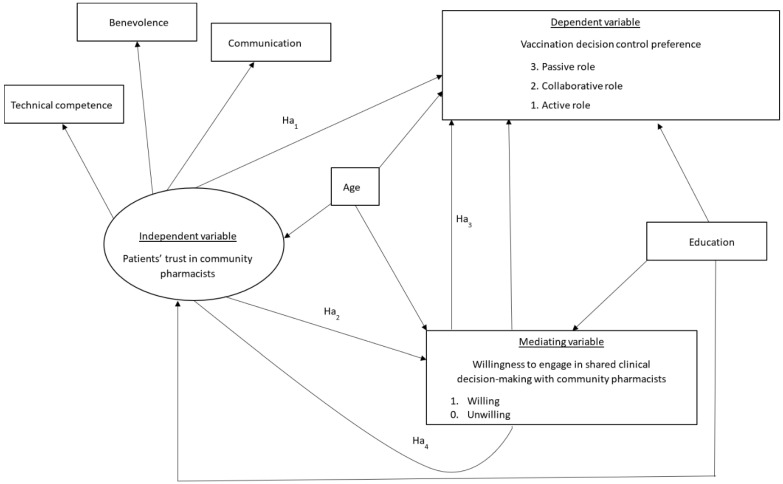
Conceptual model. The conceptual model posits that patients’ trust in community pharmacists directly influences their vaccination decision control preference and their willingness to engage in shared clinical decision-making (SCDM), with willingness mediating the relationship between trust and vaccination decision control preference. Benevolence, Technical Competence, and Communication are the three components of the Patients’ Trust in Community Pharmacists Scale used to assess trust. Vaccination decision control preference represents participants’ preferred role in decision-making about receiving a recommended vaccine: level 1 = active role (making decisions themselves), level 2 = collaborative role (making decisions together with their provider), and level 3 = passive role (providers making decisions for patients). Age and education were adjusted for as confounders in the pathways among these three variables.

Community pharmacists were chosen as the focus of this study because they are commonly visited for immunizations in the United States and have frequent opportunities to engage with patients who may be eligible for SCDM recommendations [1,18,19,20]. This study aimed to: (1) describe patients’ awareness and knowledge of vaccinations with SCDM, their willingness to engage in SCDM with pharmacists and their vaccination decision control preference; (2) examine the association between patients’ characteristics and trust in community pharmacists with their willingness to engage in the SCDM process with pharmacists; and (3) assess the structural relationship between patients’ trust in community pharmacists and their preference for vaccination decision control during SCDM, as well as how patients’ willingness to engage with pharmacists mediates this relationship. Accordingly, to fulfill the 3rd objective, four hypotheses were tested as shown in our conceptual model in Figure 1 below:

**Ha_1_:**  *Patients’ trust in community pharmacists has a direct effect on their vaccination decision control preference.*

**Ha_2_:**  *Patients’ trust in community pharmacists has a direct effect on their willingness to engage in SCDM with pharmacists.*

**Ha_3_:**  *Patients’ willingness to engage in SCDM with pharmacists has a direct effect on their vaccination decision control preference.*

**Ha_4_:**  *Patients’ trust in community pharmacists has an indirect effect on their vaccination decision control preference through their willingness to engage in SCDM with pharmacists.*

## 2. Materials and Methods

### 2.1. Study Design, Sample and Setting

This study employed a cross-sectional design, and the data presented were part of a larger study focused on COVID-19 vaccines [21]. Participants were a convenience sample recruited from Qualtrics^XM^ panel and were required to be residents of the state of Alabama in the U.S., aged 18 years and older. Alabama was chosen because of low COVID-19 vaccine uptake in the state [22]. Qualtrics^XM^ offers an online survey tool for creating and distributing surveys [23]. The company maintains a Qualtrics panel, which is a group of pre-selected individuals who can be surveyed for targeted research purposes. Quotas were set based on ethnicity, race, COVID-19 vaccination status, and residence to ensure that the final sample included individuals with diverse vaccination behaviors (i.e., those who are inclined to receive vaccines and those who are hesitant or resistant), as well as adequate representation of certain minority groups (e.g., Hispanic, American Indian, Black) and rural populations. Ethical approval was granted by the Institutional Review Board of Auburn University, where the authors are affiliated.

### 2.2. Data Collection and Measures

Data was collected from participants by Qualtrics^XM^ using an online questionnaire (Supplementary Material S1) distributed from 1 February to 11 March 2024. Of the 3951 invites sent by Qualtrics^XM^, 3101 (78%) participants responded to the survey. A total of 1020 valid and complete responses were obtained at the end of the data collection period, following the removal of 2081 responses for reasons such as data quality checks (e.g., incomplete responses, in-survey terminations (e.g., responses with incorrect ZIP codes or from non-Alabama residents), quality terminations (e.g., bot or duplicate responses) and over-quota responses terminations (those received after the sample quota had already been filled).

The main independent variable of interest was patients’ trust in community pharmacists. Participants’ characteristics were included as control variables and were assessed using open-ended questions (e.g., ‘Please indicate the 5-digit ZIP code of your primary residence’) and multiple-choice questions about the participants’ age, race, ethnicity, political affiliation, etc. Participants’ trust in community pharmacists was assessed using the 30-item validated Patient Trust in Community Pharmacists (TRUST-Ph) scale [24]. The scale consists of three components: Benevolence, Technical Competence, and Communication. Benevolence assessed the extent to which participants trust that pharmacists care about their well-being, the technical component assessed the extent to which participants believe pharmacists have the knowledge, skills, and expertise to fulfill their role, and the communication component assessed participants’ trust that pharmacists can communicate clearly and answer questions during counseling. The response options for all three components were based on a 5-point Likert scale, ranging from “strongly disagree” (1) to “strongly agree” (5). Using our dataset, all scale components showed good internal consistency, with Cronbach’s alphas ranging from 0.88 to 0.92. The items, means, standard deviations and Cronbach’s alphas for all of the components can be found in Supplementary Material Table S1.

The primary outcome variables were (1) vaccination decision control preference, which was defined as participants’ preference for who should make the final vaccination decision during the SCDM process, and (2) willingness, which was defined as participants’ willingness to engage in an SCDM conversation with community pharmacists. For the first primary outcome, participants chose their preference from four response options: (a) themselves, (b) their provider (including doctors, pharmacists and nurse practitioners) (c) they and their provider, or (d) they and their friends and/or family. Given the low number of respondents selecting the fourth option (*n* = 96) and our need to maintain statistical power, these were excluded from the analysis. The remaining three response options were treated as an ordinal variable based on the level of patient involvement and trust in providers, with “themselves” (1) representing high level of patient of involvement and lower trust in providers, “patient and provider” (2) indicating moderate patient involvement and a balanced level of trust in providers, and “provider alone” (3) representing no patient involvement and greater trust in providers [10,25,26]. The ordinal variable was used to describe participants’ preferred role in decision-making: level 1 = active role, level 2 = collaborative role, and level 3 = passive role. This classification allowed for a structured analysis of how patient trust influences decision-making preference.

The second primary outcome was assessed using a single item that required participants to indicate the extent to which they would be willing to participate in an SCDM discussion if a pharmacist were to initiate it. Responses were measured on a 6-point Likert scale, ranging from ‘totally unwilling’ (1) to ‘totally willing’ (6). For analysis, the willingness variable was operationalized as a binary variable, where totally unwilling to somewhat unwilling responses were grouped as ‘0’ and totally willing to somewhat willing responses were categorized as ‘1’. This willingness variable was used in the analyses for the second objective (as a primary outcome) and the third objective (as a mediating variable).The secondary outcomes were participants’ awareness and knowledge of vaccination recommendations based on SCDM. In the questionnaire, participants were provided with a brief contextual description of SCDM before being asked the awareness and knowledge questions. For awareness, participants were asked to indicate whether they were aware that certain vaccinations were recommended using SCDM prior to the day they completed the questionnaire. This wording helps mitigate any potential priming effects. The response options were ‘yes’ or ‘no.’ For the knowledge, participants were assessed on all SCDM recommendations at the time of the study. Specifically, they were asked to indicate which vaccination recommendations they knew to be based on SCDM from a list of seven statements that included four correct statements, and three incorrect statements (serving as plausible distractors), based on the ACIP recommendations at the time of the study. The response options for all statements were ‘yes, it is based on SCDM’ or ‘no, it is not based on SCDM. The questionnaire was pre-tested for clarity and accuracy by 15 Alabama residents who were not part of the study sample.

### 2.3. Statistical Analysis

A total of 924 participants were included in the data analysis. Frequency, percentage, median, and range were used to summarize the participant characteristics. The TRUST-Ph components were summarized using mean and standard deviation. Awareness, vaccination decision control preference, and willingness variables were summarized using frequencies and percentages. Knowledge was summarized as the percentage of participants who correctly identified whether each vaccine was or was not recommended under SCDM. To achieve the second study objective, bivariate logistic regression was used to examine associations between participant characteristics, TRUST-Ph components, and willingness. Variables significant in the bivariate analyses were then assessed for multicollinearity by calculating their variance inflation factors (VIF) using linear regression. Those without significant multicollinearity were subsequently included in a multivariable logistic regression to estimate the effect of trust on willingness while adjusting for the other control variables. SAS^®^ OnDemand for Academics Version 9.4 was used to perform the descriptive and logistic regression analyses.

For the third study objective, structural equation modeling (SEM) was used to assess the direct and indirect paths in the proposed conceptual model (Figure 1), while adjusting for two confounders, age and education, identified from the literature [10,27,28,29]. These two confounders were included because they were the most commonly reported variables in prior SCDM studies and also significant in our multivariable analyses. The SEM analysis was conducted using the Lavaan package in R statistical software Version 4.2.1, with the weighted least squares mean and variance-adjusted estimator. VIF was also used to evaluate multicollinearity between trust, willingness, age and education. Model fit was assessed using several indices, including comparative fit index (CFI), Tucker–Lewis Index (TLI), root mean square error of approximation (RMSEA), and standardized root mean square residual (SRMR). Statistical significance for all statistical tests was set at *p* < 0.05.

## 3. Results

### 3.1. Characteristics of the Study Participants (N = 924)

A greater percentage of the study participants were female (69.8%), White (70.1%), not Hispanic or Latino (93.5%), not married (58.7%), and within the 45–54 years age group (19.9%). Almost half held a high school diploma or a graduate equivalency degree (48.8%). Only 43.9% of participants were employed, and nearly 69% had a household income of $60,000 or less. Participants were divided by political party affiliation: Republican (42.3%), Independent (23.8%), Democrat (21.3%), and Other (12.6%). Less than 10% reported they had no health insurance and 41.0% of the participants resided in rural areas. Very few participants indicated they had slight or no confidence (12.6%) in understanding health information, and approximately 85% reported visiting their provider at least once in 2023 to discuss their health needs. The median number of chronic conditions or risk factors reported by participants was 1, with values ranging from 1 to 23 (see Table 1).

### 3.2. Participants’ Awareness and Knowledge of SCDM for Vaccines, Willingness to Engage in SCDM with Community Pharmacists and Vaccination Decision Control Preference (N = 924)

**Figure 2:**
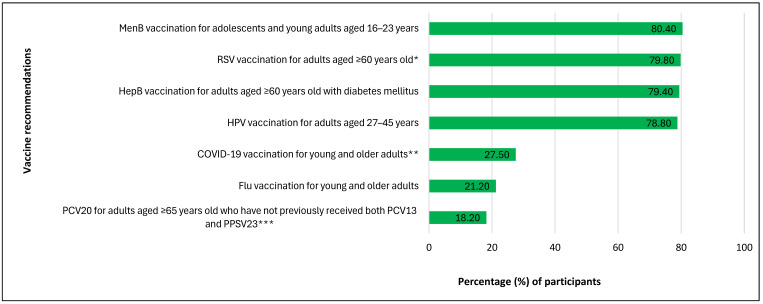
Percentage of participants aware of vaccination recommendations based on shared clinical decision-making who have correct knowledge of these recommendations (*N* = 501).* SCDM is no longer recommended for RSV vaccination as of 26 June 2024; ** COVID-19 vaccination is recommended based on SCDM for individuals who are moderately or severely immunocompromised (23 October 2024) and for all children and adolescents aged 6 months through 17 years (30 May 2025); *** PCV21 was added to the SCDM category for adults aged ≥ 65 years who have not previously received both PCV13 and PPSV23 as of 27 June 2024. The percentages represent participants who correctly identified that MenB, RSV, HepB, and HPV vaccinations for the specified groups were based on shared clinical decision-making, as well as those who correctly identified that COVID-19, flu, and PCV20 vaccinations for the specified groups were not based on shared clinical decision-making.

Nearly half (45.8%) of participants stated they were unaware that some vaccines were recommended based on SCDM (Table 2). Of those who were aware (n = 501), over 78% correctly identified MenB vaccination for adolescents and young adults aged 16–23 years, RSV vaccination for adults aged 60 years and older, HepB vaccination for adults aged 60 years and older with diabetes mellitus, and HPV vaccination for adults aged 27–45 years as based on SCDM. However, less than 30% of participants correctly identified that the recommendations for PCV20 for individuals aged ≥65 years who had not previously received PCV13 and PPSV23, flu vaccination, and COVID-19 vaccination for young and older adults were not based on SCDM (Figure 2).

The results also showed that participants’ willingness to participate in SCDM discussions varied widely (Table 2). About 60.8% of participants were willing to some extent, with the largest group being somewhat willing (31.2%). On the other hand, 39.2% of participants were unwilling to some extent, with the greatest proportion being totally unwilling (16.8%).

**Figure 3:**
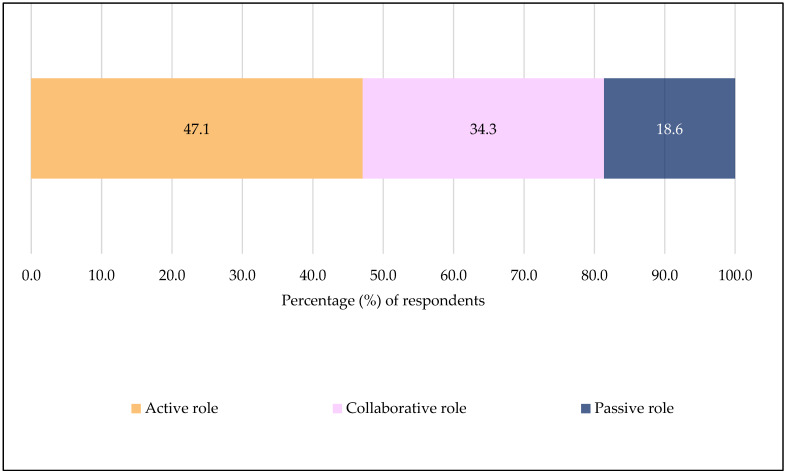
Participants’ Preferred Role in Making the Final Decision on Vaccination during the Shared Clinical Decision-making Process (*N* = 924). Level 1 = active role (making decisions themselves), level 2 = collaborative role (making decisions together with their provider), and level 3 = passive role (providers making decisions for patients).

Figure 3 shows that most participants preferred to make the decision to receive a recommended vaccine during an SCDM process themselves (47.1%) or in collaboration with their provider (34.3%). Only a small percentage (18.6%) wanted their providers to make the decision for them.

### 3.3. Factors Influencing Participants’ Willingness to Engage in Shared Clinical Decision-Making with Community Pharmacists (N = 924)

Table 3 presents the results of the bivariate and multivariable logistic regression analyses examining the relationship between the outcome variable—participants’ willingness to engage in SCDM with community pharmacists (recategorized as willing vs. unwilling) and the predictors (Trust-Ph scale components) and control variables (participant characteristics). The bivariate analyses showed that race, age, highest degree obtained, employment status, political affiliation, confidence in understanding health-related information, frequency of provider visits, and patients’ trust in community pharmacists’ communication skills, technical competence and benevolence towards their patients were significantly associated with the willingness variable. Multicollinearity diagnostics revealed no significant multicollinearity, as the VIF values for these variables ranged from 1.06 to 3.48 (Supplementary Material Table S2). VIF values > 5 to 10 typically indicate high multicollinearity [30]. The multivariable model showed that frequency of provider visits, technical competence, and employment status were not statistically significant. Specifically, Black participants were approximately 50% less likely to be willing to participate in SCDM discussions with pharmacists than White participants (AOR = 0.499, 95% CI = 0.326, 0.763). Those aged 25–34 years (AOR = 1.913, 95% CI = 1.070, 3.419) were 91% more likely to be willing to engage with pharmacists on SCDM compared with the younger age group aged 18–24 years. Compared to participants who did not complete high school, those with higher levels of education were significantly more likely to express willingness to engage in SCDM with pharmacists. Participant with a high school diploma or graduate equivalency degree were nearly three times more likely to be willing to engage (AOR = 2.855, 95% CI = 1.518, 5.369), those with associate’s degree or vocational certificate were approximately 3.5 times more likely (AOR = 3.472, 95% CI = 1.753, 6.880), and participants with a 4-year bachelor’s degree or higher were over three times more likely (AOR = 3.179, 95% CI = 1.562, 6.471). Participants who identified as members of the U.S. Democratic Party were two times more likely (AOR = 2.133, 95% CI = 1.331, 3.421) to be willing to engage in SCDM with pharmacists compared to those affiliated with the Republican Party. Additionally, confidence in understanding health information was positively associated with willingness to engage. Individuals who reported high confidence were about 66% more likely (AOR = 1.655, 95% CI = 1.052, 2.605) and those with moderate confidence were approximately twice as likely (AOR = 2.118 95% CI = 1.314, 3.416) than participants with little or no confidence. Additionally, the odds of participants being willing to engage in SCDM with community pharmacists increased by 78% for every one unit increase in trust in the community pharmacists’ benevolence (AOR = 1.780, 95% CI = 1.254, 2.524) and by 42% for every one unit increase in trust in the pharmacist’s communication skills (AOR = 1.420, 95% CI = 1.009, 1.999).

### 3.4. Structural Equation Modeling Results (N = 924)

**Figure 4:**
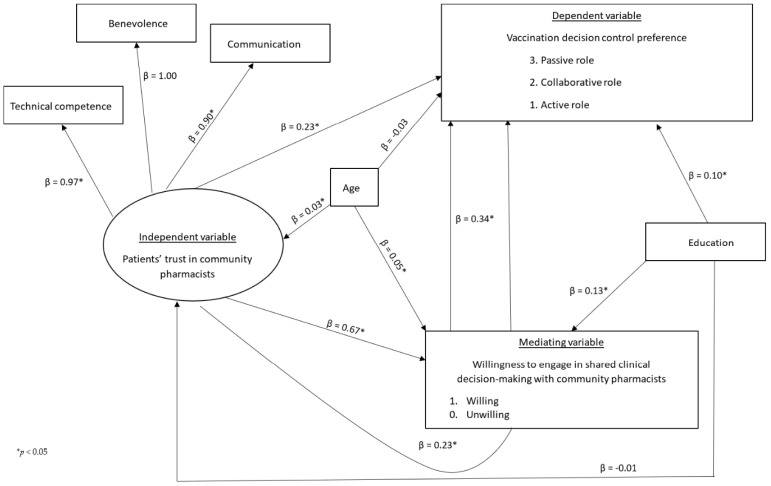
Summary of Path Analysis.

Supplementary Material Table S3 indicates no significant multicollinearity between the confounders, trust and willingness variables (VIF ranged from 1.09 to 3.41). Table 4 and the path analysis results (Figure 4) support the tested hypotheses. Trust had a significant direct and positive effect on vaccination decision control preference (β = 0.23, *p* < 0.05), meaning that for every one-point increase in trust, there was a 0.23-point increase in vaccination decision control preference. This suggests a shift toward higher values on the ordinal scale, indicating that patients with greater trust in pharmacists were more likely to prefer shared or provider-led decision-making rather than making vaccination decisions on their own. Trust also had a significant direct and positive relationship with the mediating variable, willingness. A one-point increase in trust was associated with a 0.67-point increase in willingness (β = 0.67, *p* < 0.05), indicating that patients who trust their pharmacists were more inclined to participate in SCDM. Similarly, the mediating variable had a significant direct and positive relationship with the primary outcome, decision control preference. A one-point increase in willingness to engage in SCDM was associated with a 0.34-point shift in vaccination decision control preference (β = 0.34, *p* < 0.05), moving patients away from wanting to make decisions entirely on their own toward more collaborative or provider-led approaches. The thresholds defining the cut-points between categories for willingness and decision control variables are shown in Supplementary Material Table S4. The total effect of trust on vaccination decision control preference was significant (β = 0.46, *p* < 0.05), with mediation (β = 0.23, *p* < 0.05), accounting for 50% of the total effect. Table 4 further shows that the model fit the data well, with CFI = 0.998, TLI = 0.998, RMSEA = 0.020, and SRMR = 0.014. These values meet commonly accepted thresholds for good model fit (CFI and TLI ≥ 0.95, RMSEA ≤ 0.06, SRMR ≤ 0.08) [31,32,33].

## 4. Discussion

This study highlights an awareness gap, as nearly half of participants (45.8%) were unaware that some vaccine recommendations necessitate SCDM to ascertain if the patient would benefit from the respective vaccine. This lack of awareness is problematic because patients who could benefit from vaccination may go without important discussions (which is the primary goal of SCDM) if providers do not actively initiate them, potentially resulting in missed vaccinations [34,35]. Most patients can only learn about vaccine recommendations during provider visits [36]. However, factors including limited provider knowledge of vaccine recommendations, low provider confidence in vaccine safety and effectiveness, lack of or inadequate decision support tools and systems may hinder providers’ ability to engage with patients on SCDM vaccines [14,35,37,38,39]. Further, a recent survey of physicians reported that most forecasting software displays SCDM-based vaccines as routine recommendations, without distinction [14]. When vaccines appear as routinely recommended, the default is for the providers to recommend these vaccines to patients without assessing benefits and risks through collaborative discussions, thereby not adhering to the principles of SCDM per the ACIP guidelines. Implementing clearer prompts for providers can help them differentiate SCDM vaccines from routine, ensuring that SCDM vaccines are addressed appropriately [14,37]. Additionally, staying current with the recommended vaccine schedule can help providers identify which vaccines require SCDM, even when forecasting software does not indicate it. Educational campaigns that improve providers’ knowledge of vaccine safety and effectiveness, along with awareness of decision-support tools such as the three-talk model for SCDM and CDC resources, may further increase their confidence in discussing SCDM recommendations with patients. [1,2,40]. Implementing educational campaigns targeted at patients is another effective strategy to increase awareness of SCDM vaccines, and may also encourage patients to initiate discussions about these vaccines in future encounters [41]. Such efforts may promote greater patient engagement in the SCDM process and contribute to increased patient satisfaction [10,42].

The findings also show important demographic and psychosocial characteristics that could influence willingness to engage in SCDM with a community pharmacist, such as race, age, educational attainment, political affiliation, and confidence in understanding health-related information. This study found that Black participants were approximately 50% less likely than White participants to be willing to engage in SCDM discussions with pharmacists. This may be related to factors such as the legacy of historical discrimination in healthcare, socioeconomic barriers, poor patient–clinician communication, and medical mistrust. These factors can influence access to healthcare resources, interactions with healthcare providers, and willingness to participate in pharmacist-led SCDM [43,44,45,46,47,48]. Study participants aged 25–34 years demonstrated a 91% higher inclination toward engaging in SCDM with pharmacists compared to those aged 18–24 years. This difference may be attributed to the transitional and critical nature of the 25–34 age period, during which individuals often face increased stress and responsibilities that impact their health and well-being. As a result, they tend to have greater health concerns, leading to increased healthcare engagement, more frequent communication with healthcare providers, and greater reliance on pharmacists as accessible sources of health counseling and management [49,50,51]. Additionally, compared to participants who did not complete high school, this study found that higher educational attainment was associated with a greater likelihood of engaging in SCDM with pharmacists. A similar finding reported by Pot et al., showed that higher educational levels positively correlated with SCDM interventions and improved participants’ acceptability, decisional conflict, and willingness toward HPV vaccination uptake [52]. The political affiliation of the study participants was also found to impact their willingness to engage. People affiliated with the U.S. Democratic Party had a higher likelihood to engage than Republican Party participants. This could be supported by Geana et al., whose study reported that political ideology shapes health behaviors, specifically COVID-19 preventive behaviors and vaccination, with such health behaviors originally politicized for several years [53,54]. These findings align with other evidence showing that political affiliation can also influence general vaccine attitudes [55]. Confidence level in understanding health information positively impacted SCDM engagement willingness among the study participants. This finding is supported by studies indicating that health literacy is an asset for engagement in SCDM [56,57]. 

The willingness to engage in SCDM with community pharmacists was significantly associated with higher trust in community pharmacists’ communication skills and benevolence in the multivariable analysis. SCDM relies on two essential and interconnected processes, including communication and collaboration [58]. For effective SCDM, it is essential for clinicians to have the necessary communication skills, including the ability to execute key elements of patient-centered communication [59]. In other contexts where SCDM has been employed, the communication techniques of the clinician are considered influential [60,61,62]. For example, a study of SCDM in multiple myeloma patients revealed that while trust plays a significant role in SCDM, it might be motivated by clinician communication practices showing competence, responsiveness, listening, honesty, and empathy [13]. Additionally, our findings are consistent with a study involving fertility and intensive care patients, emphasizing that benevolent behaviors, such as empathy, being attentive to both patients and their families, fostering trust, and delivering on promises, are key elements of effective SCDM [63]. Patients tend to engage more effectively when they believe their clinician is respectful and attentive to their opinions and concerns, and when they feel supported and encouraged to take part in the decision-making process [64]. These studies suggest that targeted training programs aimed at improving providers’ communication skills and benevolence may enhance patients’ trust in them. In support, structural changes in pharmacy practice, such as providing private counseling areas, implementing feedback systems (e.g., patient satisfaction surveys), and ensuring adequate staffing, may further build trust and encourage effective patient–pharmacist engagement in SCDM.

This study also shows that patients with lower trust in community pharmacists preferred to take an active (making decisions themselves) role in their vaccination decision (Figure 4). This finding aligns with a Swiss study that found that patients who prefer to have decisional control have lower trust in the healthcare team (including pharmacists) [65]. A greater percentage of participants in this study preferred to take control of their vaccination decision or make decisions together with their provider (Figure 3), which is consistent with a previous meta-analysis finding that showed that most cancer patients in the U.S. preferred an active or collaborative approach to SCDM [11]. In contrast, Canadian, Indian and Chinese cancer patients have been reported to prefer or take on a passive role to SCDM [10,11,12]. The preference for provider-led decisions may reflect a paternalistic healthcare culture in these countries where patients view doctors as authority figures and highly trust them to decide the best course of action on their healthcare without needing extensive personal involvement [10,11,26]. The preference for active or collaborative involvement in the U.S. may be explained by levels of patients’ trust in the healthcare system, increased health literacy, or increased adoption of a patient-centered approach in clinical practice [11,25,57,66]. SCDM emphasizes a collaborative approach to decision-making [1,2]. In practice, this may not always hold true due to the suggested factors. Pharmacists should consider tailoring SCDM discussions to match patients’ preferences, as this can lead to better patient satisfaction. Overall, this study shows that trust is important for the implementation of SCDM for vaccinations as intended and should not be overlooked.

One of the key strengths of this study is its use of a validated measure to assess patients’ trust in community pharmacists, which enhances the validity and reliability of our findings. Additionally, the large sample size of participants allowed for robust statistical analysis. This study also makes a significant contribution to the literature by being the first, to the best of our knowledge, to test the associations between patients’ trust in community pharmacists, their willingness to engage with pharmacists in SCDM discussions for recommended vaccines, and their vaccination decision control preferences. The findings provide new evidence on the role of trust in patient engagement in vaccine-related SCDM with pharmacists.

This study has some limitations that should be acknowledged and addressed in future research. The study participants were all recruited from the Qualtrics panel, which introduces the possibility of self-selection bias. Participants in the panel were likely those who were willing, motivated and able to participate in surveys. The study may have limited generalizability beyond Alabama. Although quota sampling was used to ensure the sample reflected the state’s demographic composition, the lack of random sampling may limit representativeness, and the focus on a single state may restrict the applicability of findings to other states within the U.S. or to other countries. The study focused on individuals aged 18 and older, while the MenB SCDM recommendations apply to those aged 16–23. The age criteria were set to allow inclusion of participants who could independently engage in SCDM conversations and make their own vaccination decisions. Excluding 16- and 17-year-olds implies that our results may not fully capture the perspectives and behaviors of this age group. Future research should consider including parents of this age group to provide a more comprehensive understanding of MenB vaccine decision-making across the full adolescent spectrum. As with survey research, this study also has the possibility of social desirability bias. Participants may have provided socially acceptable answers rather than accurate responses depicting their true knowledge, awareness, behaviors, and opinions. As with any observational study, there remains the possibility of residual confounding due to variables that were not measured or included in the analysis. While we attempted to account for key covariates known to influence the outcome, unmeasured factors such as individual-level behaviors, environmental influences, or other contextual variables may still have affected the results. The next limitation of this study is that while questions assessing willingness and decision-making preference provided insights into participants’ attitudes, they may not necessarily reflect their actual behavior in the real world. The use of a cross-sectional study design limited the analysis to identifying associations between the variables studied and did not allow for causal inferences. More robust study designs, like longitudinal and experimental designs, are recommended to establish a causal relationship.

Future research could replicate this study in other regions and care settings to strengthen the significance of the findings. Additionally, there is a need to examine how factors beyond trust, such as patient and provider vaccine confidence, SCDM communication strategies, and expectations regarding the outcomes of the SCDM process, influence patients’ willingness and vaccination decision control preference. Such insights could inform future vaccination recommendations by the ACIP of the U.S. CDC and help providers tailor discussions.

## 5. Conclusions

This study found that (1) a significant number of patients were not aware that SCDM recommendations exist for vaccines; (2) factors including patients’ race, age, education, political affiliation, confidence in understanding health information, and trust in pharmacists’ benevolence and communication skills were significantly associated with patients’ willingness to engage in SCDM with community pharmacists; and (3) patients with greater trust in community pharmacists were more likely to prefer either having their providers make vaccination decisions on their behalf or deciding collaboratively with them. Educational interventions targeting patients are needed to improve awareness and knowledge of SCDM vaccines. Tailored interventions, such as use of culturally adapted messaging by pharmacists, for subgroups with lower odds of willingness (including Black individuals, those with less than a high school diploma, individuals aged 18–24 years, Republicans, and those with low health literacy) are recommended to improve participation. Additionally, training programs for pharmacists aimed at improving their communication skills and benevolent behaviors are essential, as these may strengthen patients’ trust in pharmacists. By building and maintaining trust, pharmacists can create collaborative environments that encourage patient engagement in SCDM and support more informed vaccination choices. The study findings can inform interventions to support the implementation of SCDM for vaccines in community pharmacy settings.

## Figures and Tables

**Table 1:** Participant characteristics (*N* = 924).

Variable	*n* (%)
**Sex**	
Male	279 (30.2)
Female	645 (69.8)
**Race**	
Asian	10 (1.1)
American Indian or Alaskan Native	12 (1.3)
Black	194 (21.0)
White	648 (70.1)
Multi-racial	37 (4.0)
Other	23 (2.5)
**Ethnicity**	
Hispanic or Latino	60 (6.5)
Not Hispanic or Latino	864 (93.5)
**Age**	
18–24	104 (11.3)
25–34	134 (14.5)
35–44	172 (18.6)
45–54	184 (19.9)
55–64	149 (16.1)
65+	181 (19.6)
**Highest Degree Obtained**	
Less than high school	58 (6.3)
High school diploma or Graduate Equivalency Degree (GED)	451 (48.8)
Associate’s degree or vocational certificate	218 (23.6)
4-year bachelor’s degree or higher	197 (21.3)
**Marital Status**	
Married	382 (41.3)
Not Married	542 (58.7)
**Employment Status**	
Disabled, not able to work	143 (15.5)
Not Employed	177 (19.2)
Retired	198 (21.4)
Employed	406 (43.9)
**Household Income Level**	
$0–$30,000	344 (37.2)
$30,001–$60,000	289 (31.3)
$60,001–$90,000	131 (14.2)
$90,001–$120,000	54 (5.8)
$120,000+	56 (6.1)
I choose not to say	50 (5.4)
**Political Affiliation**	
Democrat	197 (21.3)
Independent	220 (23.8)
Republican	391 (42.3)
Other	116 (12.6)
**Insurance Status**	
Not Insured	75 (8.1)
Insured	849 (91.9)
**Residence ^a^**	
Rural	379 (41.0)
Urban	538 (58.2)
Other ^b^	7 (0.8)
**Level of confidence in understanding health-related information**	
Highly confident	516 (55.8)
Moderately confident	292 (31.6)
Slightly/not at all confident	116 (12.6)
**Frequency of healthcare provider visits regarding health concerns in 2023**	
0	143 (15.5)
1	128 (13.9)
2–4	396 (42.9)
5–7	133 (14.4)
8 or more	124 (13.4)
	Median (range)
Total number of chronic conditions	1.0 (22.0)

^a^ Rural–Urban Commuting Area Codes (RUCA) were used to determine the degree of rurality. ^b^ Missing from Zip code list.

**Table 2:** Participants’ self-reported awareness of SCDM-based vaccination recommendations and degree of willingness to engage in SCDM conversations with a community pharmacist (*N* = 924).

Variable	*n* (%)
**Awareness of SCDM vaccination recommendations**	
Not aware	423 (45.8)
Aware	501 (54.2)
**Degree of willingness to engage in SCDM with a pharmacist**	
Totally willing	131 (14.2)
Moderately willing	143 (15.5)
Somewhat willing	288 (31.2)
Somewhat unwilling	136 (14.7)
Moderately unwilling	71 (7.7)
Totally unwilling	155 (16.8)

**Table 3:** Bivariate and multivariable logistic regression of factors associated with participants’ willingness to engage in shared clinical decision-making with a community pharmacist (*N* = 924).

Factor	Effects	Unadjusted Odds Ratio (95% Confidence Interval)	*p*-Value	Adjusted Odds Ratio (95% Confidence Interval) ^a^	*p*-Value ^a^
Sex	Female vs. male	1.060 (0.795, 1.412)	0.692		
Race	Asian vs. White	0.906 (0.253, 3.242)	0.879	0.994 (0.250, 3.948)	0.994
	American Indian or Alaskan Native vs. White	1.208 (0.360, 4.054)	0.760	1.541 (0.395, 6.006)	0.534
	Black vs. White	**0.698 (0.505, 0.965)**	**0.030**	**0.499 (0.326, 0.763)**	**0.001**
	Multiracial vs. White	1.258 (0.621, 2.550)	0.524	1.433(0.643, 3.192)	0.379
	Other vs. White	1.132 (0.473, 2.710)	0.780	1.133 (0.430, 2.984)	0.800
Ethnicity	Hispanic or Latino vs. not Hispanic or Latino	1.039 (0.606, 1.779)	0.890		
Age	25–34 vs. 18–24	**1.827 (1.087, 3.071)**	**0.023**	**1.913 (1.070, 3.419)**	**0.029**
	35–44 vs. 18–24	1.353 (0.830, 2.204)	0.225	1.350 (0.774, 2.354)	.290
	45–54 vs. 18–24	**1.746 (1.074, 2.838)**	**0.025**	1.760 (0.999, 3.100)	0.050
	55–64 vs. 18–24	**2.362 (1.410, 3.957)**	**0.001**	1.760 (0.939, 3.299)	0.078
	65+ vs. 18–24	**2.380 (1.450, 3.908)**	**0.001**	1.355 (0.654, 2.806)	0.414
Highest degree obtained	High school diploma or Graduate Equivalency Degree (GED) vs. less than high school	**2.580 (1.463, 4.551)**	**0.001**	**2.855 (1.518, 5.369)**	**0.001**
	Associate’s degree or vocational certificate vs. less than high school	**3.292 (1.800, 6.019)**	**0.000**	**3.472 (1.753, 6.880)**	**0.000**
	4-year bachelor’s degree or higher vs. less than high school	**3.497 (1.897, 6.447)**	**<0.0001**	**3.179 (1.562, 6.471)**	**0.001**
Marital status	Married vs. not married	1.133 (0.866, 1.483)	0.362		
Employment status	Disabled, not able to work vs. employed	1.164 (0.788, 1.720)	0.445	1.091 (0.675, 1.766)	0.722
	Not Employed vs. employed	0.990 (0.692, 1.415)	0.954	1.090 (0.727, 1.634)	0.675
	Retired vs. employed	**1.634 (1.140, 2.343)**	**0.008**	1.365 (0.782, 2.384)	0.274
Household income level	$30,001–$60,000 vs. $0–$30,000	1.203 (0.874, 1.657)	0.257		
	$60,001–$90,000 vs. $0–$30,000	1.393 (0.916, 2.118)	0.122		
	$90,001–$120,000 vs. $0–$30,000	1.457 (0.796, 2.668)	0.222		
	$120,000+ vs. $0–$30,000	1.126 (0.632, 2.006)	0.687		
	I choose not to say vs. $0–$30,000	0.855 (0.471, 1.552)	0.607		
Political affiliation	Democrat vs. republican	**1.586 (1.100, 2.286)**	**0.014**	**2.133 (1.331, 3.421)**	**0.002**
	Independent vs. republican	1.077 (0.768, 1.511)	0.667	1.441 (0.968, 2.146)	0.072
	Other vs. republican	**0.633 (0.417, 0.960)**	**0.031**	1.085 (0.671, 1.757)	0.738
insurance	Not insured vs. insured	0.675 (0.421, 1.084)	0.104		
Residence ^b,c^	Rural vs. urban	1.018 (0.778, 1.333)	0.895		
Confidence in understanding health-related information	Highly confident vs. (low) slightly/not at all confident	**2.451 (1.626, 3.695)**	**<0.0001**	**1.655 (1.052, 2.605)**	**0.029**
	Moderately confident vs. (low) slightly/not at all confident	**2.523 (1.625, 3.917)**	**<0.0001**	**2.118 (1.314, 3.416)**	**0.002**
Frequency of healthcare provider visits regarding health concerns in 2023	1 vs. 0	1.290 (0.800, 2.080)	0.297	1.052 (0.620, 1.786)	0.850
	2–4 vs. 0	**2.017 (1.369, 2.971)**	**0.000**	1.489 (0.965, 2.297)	0.072
	5–7 vs. 0	**2.157 (1.326, 3.508)**	**0.002**	1.578 (0.913, 2.728)	0.102
	8 or more vs. 0	**2.078 (1.267, 3.406)**	**0.004**	1.395 (0.787, 2.472)	0.254
Total number of chronic conditions		1.088 (0.986, 1.199)	0.092		
Benevolence		**2.443 (1.970, 3.030)**	**<0.0001**	**1.780 (1.254, 2.524)**	**0.001**
Technical competence		**2.489 (1.973, 3.139)**	**<0.0001**	1.163 (0.765, 1.769)	0.480
Communication		**2.387 (1.916, 2.975)**	**<0.0001**	**1.420 (1.009, 1.999)**	**0.045**

^a^ Empty boxes indicate non-significant variables from the bivariate analyses that were dropped from the multivariable analysis; ^b^ Rural–Urban Commuting Area Codes (RUCA) were used to determine the degree of rurality; ^c^ *N* = 917 (excluded those whose ZIP codes did not match the ZIP code list); Bold = *p* < 0.05.

**Table 4:** Effect results and Model Fit.

Effect Type	Hypothesis	β (95% CI)	Standard Error	*p* Value
Direct effect of patient’s trust in community pharmacists on vaccination decision control preference	Ha_1_	0.23 (0.09, 0.37)	0.07	**0.001**
Direct effect of patient’s trust in community pharmacists on willingness to engage in SCDM with community pharmacists	Ha_2_	0.67 (0.54, 0.79)	0.07	**0.000**
Direct effect of willingness to engage in SCDM with community pharmacists on vaccination decision control preference	Ha_3_	0.34 (0.24, 0.43)	0.05	**0.000**
Indirect effect of patient’s trust in community pharmacists on vaccination decision control preference through willingness to engage in SCDM with community pharmacists	Ha_4_ = Ha_2_ × Ha_3_	0.23 (0.16, 0.29)	0.04	**0.000**
Total effect of patient’s trust in community pharmacists on vaccination decision control preference	Ha_1_ + Ha_4_	0.46 (0.33, 0.58)	0.06	**0.000**

Bold = *p* < 0.05; comparative fit index: 0.998; Tucker–Lewis index: 0.998; root mean square error of approximation: 0.020; standardized root mean square residual: 0.014.

## Data Availability

The study data is available upon request from the corresponding author.

## Supplementary Materials

The following supporting information can be downloaded at: https://www.mdpi.com/article/10.3390/ijerph22101525/s1, Supplementary Material S1: Study questionnaire; Table S1: Characteristics of components of Trust in Community Pharmacists (TRUST-Ph) scale; Table S2: Multicollinearity results for significant predictors identified in the bivariate logistic regression analyses; Table S3: Variance inflation factors of predictor variables in the structural equation model; Table S4: Threshold estimates for categories of Willingness and Vaccination Decision Control Preference variables.

## Abbreviations

The following abbreviations are used in this manuscript:

U.S.	United States
SCDM	Shared Clinical Decision-Making
ACIP	Advisory Committee on Immunization Practices
CDC	Centers for Disease Control and Prevention
MenB	Meningococcal B
HepB	Hepatitis B
HPV	Human Papillomavirus
PCV20	20-valent Pneumococcal Conjugate Vaccine
PCV13	13-valent Pneumococcal Conjugate Vaccine
PPSV23	23-valent Pneumococcal Polysaccharide Vaccine
RSV	Respiratory Syncytial Virus
PCV21	21-valent Pneumococcal Conjugate Vaccine
COVID-19	Coronavirus Disease 2019
TRUST-Ph	Trust in Community Pharmacists
