# Supplementary material for: From Trust to Choice: A Cross-Sectional Survey of How Patient Trust in Pharmacists Shapes Willingness and Vaccination Decision Control Preferences
Source: Int J Environ Res Public Health. 2025 Oct 5;22(10):1525. doi: 10.3390/ijerph22101525 (PMC12562420; doi:10.3390/ijerph22101525)
Supplement: Supplementary file 1 [file ijerph-22-01525-s001.zip › Supplementary Material S1 Study questionnaire.pdf]

Beliefs and Decisions to Obtain the COVID-19 Vaccines Among Alabama Residents

Introduction: This survey gathers information about your perceptions of and your intention to obtain the updated COVID-19 vaccine. It also explores your opinions regarding medical and public health officials, as well as your thoughts on shared clinical decision-making process for certain vaccines. This survey should take about 15-20 minutes.

Section I: Perceptions of COVID-19 Vaccines

Instructions. Please indicate your level of agreement with the following statements about COVID-19 vaccines.

Item	Strongly Disagree	Disagree	Neutral	Agree	Strongly Agree
1. Getting COVID-19 vaccines is a good means to protect myself from the COVID-19 disease.					
2. COVID-19 vaccines are important for my health.					
3. To be vaccinated is important for the health of others in my community.					
4. COVID-19 vaccines are effective.					
5. Generally, I do what my doctor or health care provider recommends about COVID-19 vaccines.					
6. COVID-19 vaccines can lead to severe allergic reactions such as anaphylactic shock.					
7. COVID-19 vaccinations can cause the very illness they are designed to prevent.					
8. COVID-19 vaccinations can cause blood clots and complications including thrombosis and embolism.					
9. COVID-19 vaccines overwhelm the immune system.					
10. Certain emerging diseases, like COVID-19 were strategically created and spread by the government in order to have the opportunity to develop vaccines.					
11. We have to be cautious about the recommendations that are given to the					

general public regarding the COVID-19 vaccine uptake.					
12. I have chills (goosebumps) when I think about being vaccinated with one of the COVID-19 vaccines.					
13. I have an upset stomach (nausea, butterflies) when I think about being vaccinated with one of the COVID-19 vaccines.					
14. My heart beats rapidly (change in heart rate) when I think about being vaccinated with one of the COVID-19 vaccines.					
15. I have sleeping difficulties because I'm worrying about being vaccinated with one of the COVID-19 vaccines.					

Section II: Opinions about Medical Doctors, Pharmacists, and Public Health Officials

Instructions: The following questions ask for your opinions and beliefs about general medical doctors, not necessarily your own doctors. Please think about your general impressions of medical doctors. These impressions might be based on your experiences or things you have heard from family, friends, or the media.

Please indicate **how much you disagree or agree with each of the statements**.

Item	Strongly Disagree	Disagree	Neither Agree nor Disagree	Agree	Strongly Agree
1. Doctors have good judgement.					
2. Doctors explain the benefits and risks of treatments to patients.					
3. Doctors listen to patients.					
4. Doctors believe patients when they say something is wrong.					
5. Doctors follow up with patients when needed.					
6. Doctors put making money above patient needs.					
7. Doctors recommend expensive treatments to make money.					
8. Doctors hide mistakes.					

9. Doctors might experiment on patients without their knowledge.					
10. Doctors rush through appointments.					
11. Doctors are held accountable if they make a mistake.					
12. Doctors are held accountable if they treat patients unfairly.					
13. Doctors are held accountable if they discriminate against patients.					
14. Doctors keep medical records private.					
15. Doctors use secure systems to store medical records.					
16. Doctors respect patient privacy.					
17. Doctors treat patients fairly, regardless of their ability to pay.					
18. Doctors treat patients of all races and ethnicities fairly.					
19. Doctors treat patients fairly, regardless of their gender (e.g., male, female, or nonbinary).					
20. Doctors treat patients fairly, regardless of their sexual orientation (e.g., straight, gay, lesbian, or bisexual).					
21. Doctors treat patients fairly, regardless of their weight.					
22. Doctors treat patients fairly, regardless of their religion.					
23. Doctors treat patients fairly, regardless of their education level.					
24. Doctors treat patients with a history of mental illness unfairly.					
25. Doctors treat patients diagnosed with HIV unfairly.					
26. Doctors treat patients who abuse drugs unfairly.					
27. All things considered, I trust doctors.					
28. I put my trust in doctors.					

29. Doctors are trustworthy.					

Instructions: The following questions ask for your opinions and beliefs about pharmacists in general, not necessarily your own pharmacists. Please think about your general impressions of pharmacists. These impressions might be based on your experiences or things you have heard from family, friends, or the media.

Please indicate how much you disagree or agree with each of the statements

Item	Strongly Disagree	Disagree	Neutral	Agree	Strongly Agree
1. Pharmacists do not do anything illegal or unethical.					
2. Pharmacists care about your health.					
3. Pharmacists never mislead you about anything.					
4. Pharmacists keep your sensitive medical information private.					
5. Pharmacists care about you more than their own personal benefits.					
6. Pharmacists would admit if a mistake was made when dispensing.					
7. Pharmacists put your health above the drug costs (profits).					
8. Pharmacists pay attention to your problems.					
9. Pharmacists sincerely serve you.					
10. Pharmacists put your benefits as first priority.					
11. You can tell pharmacists anything.					
12. Pharmacists choose the best treatment or medications for you.					
13. Pharmacists put highest efforts on decision about your medications.					
14. Pharmacists are experts about drugs.					
15. Pharmacists should be the persons who make decision about your medications.					
16. Pharmacists correctly notify you how to use drugs.					

17. Pharmacists can help you with your illness.					
18. Pharmacists can solve your medication problems.					
19. Pharmacists carefully dispense your medications.					
20. You are confident in pharmacists' dispensing.					
21. Pharmacists offer you good quality medications.					
22. What pharmacists tell you is always right.					
23. You understand what pharmacists explain about how to use drugs.					
24. Pharmacists provide you opportunity to ask questions.					
25. Pharmacists clearly write medication labels.					
26. You are confident in pharmacists' counseling.					
27. Pharmacists are willing to talk or answer your questions.					
28. You always follow pharmacists' advice.					
29. Pharmacists use easy language for counseling.					
30. Pharmacists suggest you to see a doctor when needed.					

Instructions. The following questions ask for your **opinions and beliefs about public health authorities such as local and state health departments**, the **Centers for Disease Control and Prevention (CDC)**, the **Food and Drug Administration (FDA)**, and other **government health agencies**. Please think about your general impressions of public health authorities. These impressions might be based on your experiences or general beliefs about public health authorities.

Please indicate **how much you disagree or agree with each of the statements**.

Item	Strongly Disagree	Disagree	Agree	Strongly Agree
1. They do everything they should to protect the health of the population.				

2. They keep trying the same things to help the public, even when they don't work very well.				
3. They base recommendations on the best available science.				
4. They are more concerned about some racial and ethnic groups than other groups.				
5. They are concerned about all people, without caring about who has more or less money.				
6. They accurately inform the public of both health risks and benefits of medicines.				
7. They make unhelpful recommendations.				
8. They believe in what they recommend for the public.				
9. They are partly responsible for the illegal drug problems in this country.				
10. They use resources well.				
11. They waste money on health problems.				
12. They come up with new ideas to solve health problems.				
13. They ensure the public is protected against diseases.				
14. They quickly help the public with health problems.				

Section III: COVID-19 Infection and Vaccinations

Instructions: Please select the best answer for the following questions.

1) Have you <u>ever</u> received any COVID-19 vaccines?
    a. Yes
        1. Since the start of the COVID-19 pandemic, how many injections of COVID-19 vaccines have you ever received?
            1. 1 injection
            2. 2 injections
            3. 3 injections
            4. 4 injections
            5. 5 injections or more
        2. In which year did you receive the most recent COVID-19 vaccine?

1. 2021
2. 2022
3. 2023
4. 2024

3. The updated 2023 – 2024 COVID-19 vaccine was launched in September 2023. Have you received the updated 2023 - 2024 COVID-19 vaccine?
    1. Yes >>> What encouraged you to get the updated COVID-19 vaccine? >>> Go to Q5
    2. No >>> Go to Q2
    3. Unsure >>> Go to Q2

b. No >>> Go to Q2

2) The updated 2023 – 2024 COVID-19 vaccine was launched in September 2023. Do you intend to receive the updated 2023 - 2024 COVID-19 vaccine?
    a. Yes

        In which time frame do you intend to receive the updated 2023 - 2024 COVID-19 vaccine?
        1. Within the next month
        2. Within the next three months
        3. Within the next six months

    b. No

    c. Unsure

3) What concerns you the most about getting the updated 2023-2024 COVID-19 vaccine?

4) What or what circumstance would encourage you to get the updated 2023-2024 COVID-19 vaccine?

5) Since the start of the COVID-19 pandemic, have you ever been diagnosed with a COVID-19 infection that was confirmed by a test carried out by a qualified healthcare professional or an at-home COVID-19 test kit?
    a. Yes
        1. Since the start of the COVID-19 pandemic, how many times have you got COVID-19 infections that were confirmed by a test carried out by a qualified healthcare professional or an at-home COVID-19 test kit?
            a. One time
            b. Two times
            c. Three times
            d. Four or more

2. Thinking back to <u>the most recent time you had a COVID-19 infection</u>, what course of action did you take to treat your COVID-19 symptoms?
    a. I recovered on my own without consulting a healthcare professional
    b. I consulted a healthcare professional
    c. I required a hospitalization
3. Thinking back to <u>the most severe COVID-19 infection episode</u>, what course of action did you take treat your COVID-19 symptoms?
    a. I recovered on my own without consulting a healthcare professional
    b. I consulted a healthcare professional
    c. I required a hospitalization

b. No

6) Do you have a close family member or friend who has been hospitalized or died from COVID-19 infection or its complications?
    1. Yes
    2. No
    3. Unsure

Section IV: Shared Clinical Decision-Making for Certain Vaccines

Certain vaccines recommended by the Centers for Disease Control and Prevention (CDC) are not for everyone in a particular age group or everyone in an identifiable risk group. For these vaccines, **shared clinical decision-making** process is used where patients and their healthcare providers engage in a discussion to make personalized vaccination decisions, considering the patient's preferences, needs, as well as the risks and benefits of the vaccines.

1. Before today, were you aware that certain vaccines are recommended based on shared clinical decision-making?
    a) Yes
    b) No

2. Please indicate whether the CDC recommendation for each of the vaccines below is based on shared clinical decision-making.
    I. Meningococcal B (MenB) vaccines for adolescents and young adults aged 16–23 years.
        a. Yes, it is based on shared clinical decision-making
        b. No, it is not based on shared clinical decision-making

II. COVID-19 vaccines for young and older adults
   a. Yes, it is based on shared clinical decision-making
   b. No, it is not based on shared clinical decision-making

III. Hepatitis B (HepB) vaccines for adults aged 60 years and older with diabetes mellitus
   a. Yes, it is based on shared clinical decision-making
   b. No, it is not based on shared clinical decision-making

IV. Human papillomavirus (HPV) vaccination for adults aged 27–45 years.
   a. Yes, it is based on shared clinical decision-making
   b. No, it is not based on shared clinical decision-making

V. Pneumococcal conjugate vaccination (PCV20) for adults aged 65 years and older who have not previously received both PCV13 and Pneumococcal Polysaccharide Vaccine 23
   a. Yes, it is based on shared clinical decision-making
   b. No, it is not based on shared clinical decision-making

VI. Respiratory syncytial virus (RSV) vaccination for adults aged 60 years and older
   a. Yes, it is based on shared clinical decision-making
   b. No, it is not based on shared clinical decision-making

VII. Flu vaccines for young and older adults
   a. Yes, it is based on shared clinical decision-making
   b. No, it is not based on shared clinical decision-making

3. If a doctor initiates a shared clinical decision-making process about a certain vaccine with you, how willing would you be to engage in the shared clinical decision-making conversation?
   a) Totally unwilling
   b) Moderately unwilling
   c) Somewhat unwilling
   d) Somewhat willing
   e) Moderately Willing
   f) Totally willing

4. If a pharmacist initiates a shared clinical decision-making process about a certain vaccine with you, how willing would you be to engage in the shared clinical decision-making conversation?

g) Totally unwilling
h) Moderately unwilling
i) Somewhat unwilling
j) Somewhat willing
k) Moderately willing
l) Totally willing

5. Who would you most rely upon when making the final decision about getting vaccinated during the shared clinical decision-making process?
    a) Yourself >>> skip to Q6
    b) Your healthcare provider including doctors, pharmacists, nurse practitioners >>> skip to Q7
    c) Together between you and your healthcare provider >>> skip to Q8
    d) You and another trusted individual such as family members and friends >>> skip to Q9

6. Please describe why you would prefer to make the decision yourself-----------------------------
7. Please describe why you would prefer your healthcare provider to make the decision for you-----------------------------
8. Please describe why you would prefer to make the decision together with your healthcare provider -----------------------------
9. Please describe why you would prefer to make the decision together with another trusted individual -----------------------------

10. How confident are you in your ability to understand health-related information?
    a. Not at all Confident
    b. Slightly Confident
    c. Moderately Confident
    d. Very Confident
    e. Totally Confident

Section V: Your General Health and Demographic Information

Instructions: Please select the best answer for the following questions.

1. Please indicate your sex at birth.
    a. Male
    b. Female
2. Please indicate your race. Select all that apply.
    a. White
    b. Black or African-American

    c. Asian
    d. Native Hawaiian or Other Pacific Islander
    e. American Indian or Alaskan Native
    f. Other. Please specify.
3. Please indicate your ethnicity.
    a. Hispanic or Latino
    b. Not Hispanic or Latino
4. Please indicate your age at your last birthday.
    a. 18-24
    b. 25-34
    c. 34-44
    d. 45-54
    e. 55-64
    f. 65+
5. Please indicate the highest degree or level of school you completed.
    a. Less than high school
    b. High school diploma or Graduate Equivalency Degree or GED, or another diploma-equivalent alternate credential
    c. Associates Degree or Vocational Certificate
    d. 4-year Bachelor's Degree or Higher
6. Please indicate the 5-digit ZIP code of your primary residence.
7. Please indicate your marital status.
    a. Currently married
    b. Not currently married
8. Which of the following categories best de scribes your employment status?
    a. Employed
    b. Not employed
    c. Retired
    d. Disabled, not able to work
9. What is the total annual household income, from all sources?
    a. $0 - $30,000
    b. $30,001 - $60,000
    c. $60,001 - $90,000
    d. $90,001 - $120,000
    e. $120,001 +
    f. I choose not to say.
10. Are you **currently** covered by any of the following types of health insurance or health coverage plans? Select all that apply.
    a. Insurance through a current or former employer or union (of you or another family member)

    b. Insurance purchased directly from an insurance company (by you or another family member)
    c. Medicare, for people 65 and older, or people with certain disabilities
    d. Medicaid, Medical Assistance, or any kind of government-assistance plan for those with low income or a disability
    e. TRICARE or other military health care
    f. Any other type of health insurance or health coverage plan. Please specify.
11. A "caregiver" provides care for another person who needs help taking care of themselves. Are you currently a caregiver to a person(s) 65 or older?
    a. Yes
    b. No
12. Generally speaking, do you think of yourself as a Republican, a Democrat, an Independent, or something else?
    a. Republican
    b. Democrat
    c. Independent
    d. Something else

Instructions. Please select the best answer that represents your medical history.

1) Have you received a flu shot in 2023-2024?
    a. Yes
    b. No
    c. Unsure
2) In general, how would you rate your overall health now?
    a. Excellent
    b. Very Good
    c. Good
    d. Fair
    e. Poor
3) In 2023, how many times have you seen a healthcare provider including physicians, nurse practitioners, and physician assistants regarding your health concerns?
    a. None
    b. One
    c. 2 – 4
    d. 5 - 7
    e. 8 or more
4) Do you have any of the following conditions or risk factors? Select all categories that apply.
    a. Asthma
    b. Cancer
    c. Cerebrovascular disease

d. Chronic kidney disease
e. Chronic lung diseases
f. Chronic liver diseases
g. Cystic Fibrosis
h. Diabetes
i. Heart conditions
j. HIV or Human Immunodeficiency Virus
k. Mental health conditions
l. Dementia
m. Overweight or Obese
n. Physical inactivity
o. Pregnancy or recent pregnancy
p. Primary immunodeficiencies
q. Smoking, current and former
r. Solid organ or blood cell transplants
s. Tuberculosis
t. Use of corticosteroids or immunosuppressive medication
u. Sickle cell disease
v. Substance use disorders
w. Any chronic cognitive, mental, or physical disability that significantly impairs your daily functions that was NOT already listed
x. I do not have any chronic conditions or any risk factor listed above

You have finished the questionnaire. Thank you for your time and participation.
