# Supplementary material for: From Trust to Choice: A Cross-Sectional Survey of How Patient Trust in Pharmacists Shapes Willingness and Vaccination Decision Control Preferences
Source: Int J Environ Res Public Health. 2025 Oct 5;22(10):1525. doi: 10.3390/ijerph22101525 (PMC12562420; doi:10.3390/ijerph22101525)
Supplement: Supplementary file 1 [file ijerph-22-01525-s001.zip › Supplementary Tables_Table S1_ S2_ S3_ S4.pdf]

Table S1: Characteristics of components of trust in community pharmacists (TRUST-Ph) scale (*N* =924)

TRUST-Ph scale component[a]	Items	Mean (±SD)	Cronbach's α
Benevolence		3.32 (± 0.70)	.923
	1) Pharmacists do not do anything illegal or unethical		
	2) Pharmacists care about your health		
	3) Pharmacists never mislead you about anything.		
	4) Pharmacists keep your sensitive medical information private		
	5) Pharmacists care about you more than their own personal benefits		
	6) Pharmacists would admit if a mistake was made when dispensing		
	7) Pharmacists put your health above the drug costs (profits)		
	8) Pharmacists pay attention to your problems		
	9) Pharmacists sincerely serve you		
	10) Pharmacists put your benefits as first priority		
	11) You can tell pharmacists anything		
	12) Pharmacists choose the best treatment or medications for you		
Technical competence		3.45 (± 0.65)	.878
	1) Pharmacists put highest efforts on decision about your medications		
	2) Pharmacists are experts about drugs		
	3) Pharmacists should be the persons who make decision about your medications		
	4) Pharmacists correctly notify you how to use drugs		
	5) Pharmacists can help you with your illness		
	6) Pharmacists can solve your medication problems		
	7) Pharmacists carefully dispense you medications		
	8) You are confident in pharmacists' dispensing		
	9) Pharmacists offer you good quality medications		
	10) What pharmacists tell you is always right		
Communication		3.76 (± 0.68)	.913
	1) You understand what pharmacists explain about how to use drugs		
	2) Pharmacists provide you opportunity to ask questions		
	3) Pharmacists clearly write medication labels		
	4) You are confident in pharmacists' counseling		
	5) Pharmacists are willing to talk or answer your questions		
	6) You always follow pharmacists' advice		
	7) Pharmacists use easy language for counseling		
	8) Pharmacists suggest you to see a doctor when needed		

[a]Scales ranged from 1 = strongly disagree to 5 = strongly agree.

Table S2: Multicollinearity results for significant predictors identified in the bivariate logistic regression analyses (N = 924)

**Variable**	**Variance inflation factor (VIF)**
Race	1.11
Age	1.55
HIghest degree obtained	1.15
Employment status	1.37
Political affiliation	1.13
Confidence in understanding health-related information	1.06
Frequency of healthcare provider visits regarding health concerns in 2023	1.13
Benevolence	2.79
Technical competence	3.48
Communication	2.50

Table S3: Variance inflation factors of predictor variables in the structural equation model (N = 924)

**Predictor**	**Variance inflation factor (VIF)**
Willingness to engage in SCDM conversation with a community pharmacist	1.12
Benevolence	2.77
Technical competence	3.41
Communication	2.47
Age	1.11
Education	1.09

Table S4: Threshold estimates for categories of Willingness and Vaccination Decision Control Preference variables (*N* = 924)

Variable	Effect type	β (95% CI)	Standard error	*P* value
Willingness to engage with a pharmacist (mediator)	Unwilling	0.33 (0.05, 0.62)	0.14	0.020*
Vaccination decision control preference (outcome)	Active role	0.31 (0.05, 0.57)	0.13	0.018*
	Collaborative role	1.28 (1.02, 1.54)	0.13	0.000*

*$p < 0.05$

The SEM estimated thresholds based on an underlying latent continuum, with thresholds defining the cut-points between categories for both the mediating and outcome variables.
